# Assessment of Multiple Prognostic Scores in Patients With Metastatic Renal Cell Carcinoma Receiving First‐Line, Immune‐Based Combinations

**DOI:** 10.1002/cnr2.70604

**Published:** 2026-06-16

**Authors:** Hiromichi Sakurai, Masanobu Shiga, Shuya Kandori, Shota Takahashi, Akifumi Omiya, Tomoki Ishida, Kotoe Matsuda, Bunpei Isoda, Bryan J. Mathis, Manabu Komine, Masahiro Iinuma, Akira Joraku, Hiromitsu Negoro, Masakazu Tsutsumi, Takamitsu Inoue, Jun Miyazaki, Hiroyuki Nishiyama

**Affiliations:** ^1^ Department of Urology Institute of Medicine, University of Tsukuba Tsukuba Ibaraki Japan; ^2^ Department of Urology Ibaraki Prefectural Central Hospital Mito Ibaraki Japan; ^3^ Department of Urology Hitachi General Hospital Hitachi Ibaraki Japan; ^4^ Department of Cardiovascular Surgery Institute of Medicine, University of Tsukuba Tsukuba Ibaraki Japan; ^5^ Department of Urology Tsukuba Medical Center Hospital Tsukuba Ibaraki Japan; ^6^ Department of Urology National Hospital Organization Mito Medical Center Mito Ibaraki Japan; ^7^ Department of Urology International University of Health and Welfare Narita Hospital Narita Chiba Japan

**Keywords:** biomarkers, carcinoma, first‐line, immune checkpoint inhibitor, prognosis, prognostic score, renal cell

## Abstract

**Background:**

Combination immunotherapy is widely used as first‐line treatment for metastatic renal cell carcinoma (mRCC), but pretreatment prognostic stratification remains insufficiently established.

**Aims:**

To evaluate the performance of various prognostic scores in the first‐line treatment of metastatic renal cell carcinoma (mRCC) with combination immunotherapy.

**Methods and Results:**

We retrospectively analyzed 145 patients who started first‐line combination immunotherapy at six institutions from December 2015 to February 2025. We calculated IMDC, LIPI, RMH, PMHI, GRIm, C‐PLAN, mGPS, and Meet‐URO scores and performed survival (PFS, OS) and ROC analyses (PD, ORR). Survival analyses also evaluated the prognostic ability of each score by concordance index. At a median follow‐up period of 28.8 months, the median progression‐free survival (PFS) was 35.1 months and the median overall survival (OS) was not reached. Kaplan‐Meier analysis showed significant differences for all scores except between IMDC and C‐PLAN (PFS) and IMDC and mGPS (OS). Among these, PMHI and RMH demonstrated superior results in the C‐index. ROC analysis showed no score had prognostic value for PD or ORR.

**Conclusion:**

PMHI and RMH may be useful prognostic scores for survival outcomes in mRCC patients treated with immunotherapy. However, their ability to predict treatment efficacy (ORR and PD) is limited and further research is needed.

## 1 Introduction

Combination immunotherapy using immune checkpoint inhibitors (ICIs) and tyrosine kinase inhibitors (TKIs) is widely used in the treatment of metastatic renal cell carcinoma (mRCC). Recently, various combination immunotherapies formulated according to National Comprehensive Cancer Network (NCCN) guidelines, such as nivolumab plus ipilimumab (ICI‐ICI), pembrolizumab plus axitinib, nivolumab plus cabozantinib, and pembrolizumab plus lenvatinib (ICI‐TKI), are first‐line mRCC treatments [1]. However, clear criteria for regimen selection (except for the International Metastatic RCC Database Consortium [IMDC] risk classification) are scarce, and treatment efficacy varies widely. Therefore, biomarkers that can predict treatment efficacy and prognosis are increasingly needed.

Previous studies have reported that C‐reactive protein (CRP) is a biomarker for mRCC treated with combination immunotherapy. In particular, early CRP kinetics are reported as a prognostic marker in mRCC [2, 3]. However, CRP cutoff levels and measurement timing vary among reports while standards remain unestablished.

There are reports evaluating the prognostic performance of scores derived from combined clinical factors in other advanced solid cancers treated with ICI therapy [4]. Blood biomarker scores, such as the Lung Immune Prognostic Index (LIPI), showed prognostic ability but only ambiguous associations with treatment response, remaining unestablished similarly to mRCC [5].

In mRCC, the IMDC risk classification [6] is widely used as a prognostic model; however, under current standard treatment, further evaluation of other prognostic models is required. There are several new prognostic scores reported, such as the modified Glasgow Prognostic Score (mGPS) based on CRP and albumin [7], and the Meet‐URO score, which combines neutrophil‐to‐lymphocyte ratio (NLR) and bone metastases with IMDC [8]. Additionally, there are prognostic scores used in ICI therapy and phase 1 trials for other advanced solid cancers [4] but cross‐sectional comparisons of these in mRCC are limited. In addition to these clinical factors, the search for molecular biomarkers is also ongoing [9, 10].

Therefore, in this study, we evaluated the prognostic performance of various prognostic scores derived from peripheral blood parameters and tumor burden assessed before the start of combination immunotherapy as a first‐line treatment for mRCC.

## 2 Materials and Methods

### 2.1 Study Design and Patients

This was a multicenter, retrospective cohort study conducted at six hospitals, including the University of Tsukuba Hospital and its five affiliated institutions (Ibaraki Prefectural Central Hospital, Hitachi General Hospital, Tsukuba Medical Center Hospital, National Hospital Organization Mito Medical Center, and International University of Health and Welfare Narita Hospital).

We selected 165 patients with histologically diagnosed mRCC that started first‐line combination immunotherapy (ICI‐ICI or ICI‐TKI) from December 2015 to February 2025. Of 165 consecutive patients during the study period, those with missing data required for prognostic score calculation were excluded. After excluding 20 patients, 145 cases were included in the final analysis. The patient selection process is shown in Figure S2. This study was conducted with the approval of the University of Tsukuba Hospital Ethics Committee (Approval No. R05‐057, August 25, 2023) and informed consent was waived due to the study's retrospective nature.

### 2.2 Definition and Evaluation Methods

Clinical and laboratory data at the start of treatment were collected retrospectively from the electronic medical records of each institution. Histologic subtype was confirmed based on the pathological records at each participating institution. In most cases, histologic subtype was determined from primary renal tumor specimens obtained by either needle biopsy or nephrectomy. Some patients had undergone prior surgical interventions, including nephrectomy, partial nephrectomy, and metastasectomy, before starting first‐line combination immunotherapy. The selection of first‐line treatment regimens was left to the discretion of the treating physicians at each institution. PFS was defined as the period from the start of treatment to the earlier of disease progression or death, while OS was defined as the period from the start of treatment to the date of death. The objective response rate (ORR) was defined as the sum of complete response (CR) and partial response (PR). Radiological data were evaluated based on Response Evaluation Criteria in Solid Tumors (RECIST) version 1.1.

We evaluated several established prognostic scores that have been reported for mRCC and immunotherapy‐treated solid tumors. The prognostic scores were the IMDC risk classification [6], LIPI [11], the Royal Marsden Hospital (RMH) score [12], the Princess Margaret Hospital Index (PMHI) [13], the Gustave Roussy Immune (GRIm) [14] score, the C‐PLAN index [15], the mGPS [16], and the Meet‐URO score [17]. Regarding the Meet‐URO score, due to our sample size limitations (*N* = 145), we used a three‐group risk classification based on a recent validation study [18, 19]. Scoring methods and risk classifications are shown in Table 1.

**TABLE 1:** Definitions of each score and risk classification.

Score	Factor	Points	Risk group
IMDC	Hemoglobin < LLN	+1	Good: 0, Intermediate: 1–2, Poor: 3–6
	Corrected calcium > ULN	+1	
	Neutrophils > ULN	+1	
	Platelets > ULN	+1	
	KPS < 80%	+1	
	Time from diagnosis to treatment < 1 year	+1	
LIPI	dNLR > 3	+1	Good:0, Intermediate:1, Poor:2
	LDH > 250 U/L	+1	
RMH	LDH > 250 U/L	+1	Good:0, Intermediate:1, Poor:≥ 2
	Alb < 3.5 g/dL	+1	
	Metastatic organs > 2	+1	
PMHI	Alb < 3.5 g/dL	+1	Good:0, Intermediate:1, Poor:≥ 2
	Metastatic organs > 2	+1	
	ECOG PS ≥ 1	+1	
GRIm	NLR > 6	+1	Good:0, Intermediate:1, Poor:≥ 2
	LDH > 250 U/L	+1	
	Alb < 3.5 g/dL	+1	
C‐PLAN	CRP ≥ 1.0 mg/dL	+1	Good: 0–1, Poor: 2–5
	ECOG PS ≥ 2	+1	
	LDH ≥ 223 U/L	+1	
	Alb < 3.5 g/dL	+1	
	dNLR ≥ 3	+1	
mGPS	CRP > 1.0 mg/dL	+1	Good: 0, Intermediate: 1^a^, Poor: 2
	Alb < 3.5 g/dL	+1^a^	
Meet‐URO3	Bone metastases	+1	Good: 0–3, Intermediate: 4–8, Poor: 9
	NLR ≥ 3.2	+2	
	IMDC intermediate	+3	
	IMDC poor	+6	

Abbreviations: Alb: albumin; CRP: C‑reactive protein; dNLR: derived neutrophil‑to‑lymphocyte ratio; ECOG PS: Eastern Cooperative Oncology Group performance status; KPS: Karnofsky performance status; LDH: lactate dehydrogenase; LLN: lower limit of normal; NLR: neutrophil‑to‑lymphocyte ratio; ULN: upper limit of normal.

^a^ A low albumin level alone does not count as 1 point. Only when CRP is elevated and albumin is normal, it counts as 1 point and is classified as intermediate risk.

### 2.3 Statistical Analyses

The Kaplan–Meier method was used for survival analysis and log‐rank tests were performed. The Cox regression analysis was performed to compare each group and derive hazard ratios and 95% confidence intervals.

To evaluate the discriminative ability of each score for PFS and OS, Harrell's concordance index (C‐index) was used. Associations of each score with treatment response, including PD and ORR, were evaluated using receiver operating characteristic (ROC) analysis and the area under the curve (AUC) was calculated. For each score, changes among risk groups were evaluated using the Cochran‐Armitage trend test for CR and PD.

As a supplementary analysis, patient characteristics were compared between the ICI‐ICI group and the ICI‐TKI group using the Wilcoxon rank‐sum test for age and Fisher's exact test for categorical variables. Additionally, multivariable Cox regression analyses adjusted for age, sex, and treatment regimen were performed; C‐index for PFS and OS was also assessed separately in the ICI‐ICI group and the ICI‐TKI group; and time‐dependent ROC analyses for PFS and OS at 12, 36, and 60 months were conducted. Separate exploratory subgroup analyses were performed in both the intermediate‐risk and poor‐risk IMDC groups. For these IMDC‐defined subgroups, the prognostic performance of each score was assessed by the C‐index for PFS and OS.

All statistical analyses were performed using R version 4.4.3, and the significance level was set as *p* < 0.05.

## 3 Results

### 3.1 Patient Characteristics

A total of 145 patients were analyzed and patient characteristics are summarized in Table 2. The median age at treatment initiation was 70 years and approximately 80% of patients were male. More than 70% of patients were ECOG performance status (PS) 0, suggesting an overall good general condition for a majority of analyzed cases. In the IMDC risk classification, Intermediate was the most common at 63.4%, followed by Poor (22.8%) and Favorable (13.8%). The number of metachronous and synchronous cases were nearly equal. The most common sites of metastasis were the lungs, followed by lymph nodes, bones, liver, and brain. Treatment regimens were ICI‐ICI in 42.8% of cases and ICI‐TKI in 57.2% of cases. Prior surgical interventions, such as prior nephrectomy, partial nephrectomy, and prior metastasectomy, were performed in 53 (36.6%), 16 (11.0%), and 22 (15.2%) cases, respectively. Most prior metastasectomies were performed for metachronous recurrence after nephrectomy. Patient characteristics by treatment regimen (ICI‐ICI vs. ICI‐TKI) are shown in Table S1.

**TABLE 2:** Patient characteristics at the start of first‐line combination immunotherapy.

	Overall (*n* = 145)
Age at treatment initiation, median [IQR]	70 [64–75]
Sex	
Male	117 (80.7%)
Female	28 (19.3%)
ECOG PS (ECOG performance status)	
0	105 (72.4%)
≥ 1	40 (27.6%)
IMDC risk	
Favorable	20 (13.8%)
Intermediate	92 (63.4%)
Poor	33 (22.8%)
Pathology	
Clear	124 (85.5%)
Others	21 (14.5%)
Timing of metastasis	
Synchronous	78 (53.8%)
Metachronous	67 (46.2%)
Metastatic site	
Lung	96 (66.2%)
Brain	5 (3.4%)
Liver	10 (6.9%)
Bone	37 (25.5%)
Lymph node	44 (30.3%)
Treatment regimen	
Ipilimumab+Nivolumab	62 (42.8%)
Lenvatinib+Pembrolizumab	21 (14.5%)
Cabozantinib+Nivolumab	37 (25.5%)
Axitinib+Avelumab	10 (6.9%)
Axitinib+Pembrolizumab	15 (10.3%)
Prior surgical intervention	
Prior nephrectomy	53 (36.6%)
Prior partial nephrectomy	16 (11.0%)
Prior metastasectomy	22 (15.2%)

Abbreviations: IMDC: International Metastatic Renal Cell Carcinoma Database Consortium; IQR: Interquartile range; PS: performance status.

### 3.2 Distribution of the Prognostic Groups

**FIGURE 1:**
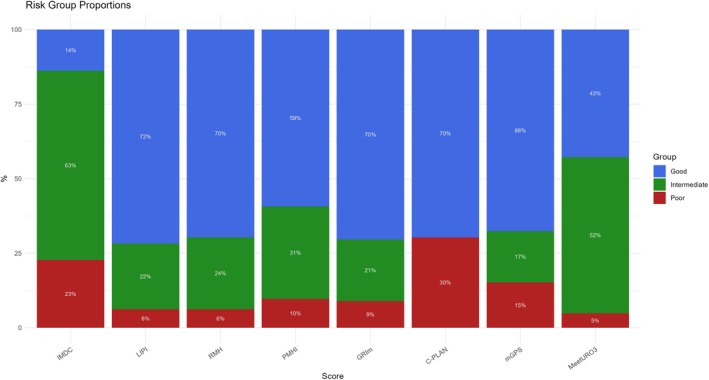
Distribution of risk groups by each prognostic score. Distributions were determined by risk classification of eight prognostic scores and stratified into Good (blue), Intermediate (green), or Poor (red).

The distribution of each prognostic score by classification is shown in Figure 1. For almost all scores, good groups were the majority (approximately 60%–70%) while in the IMDC and Meet‐URO3 scoring, the intermediate risk group was the most frequent.

### 3.3 Correlation of Prognostic Scores With PD and ORR

**FIGURE 2:**
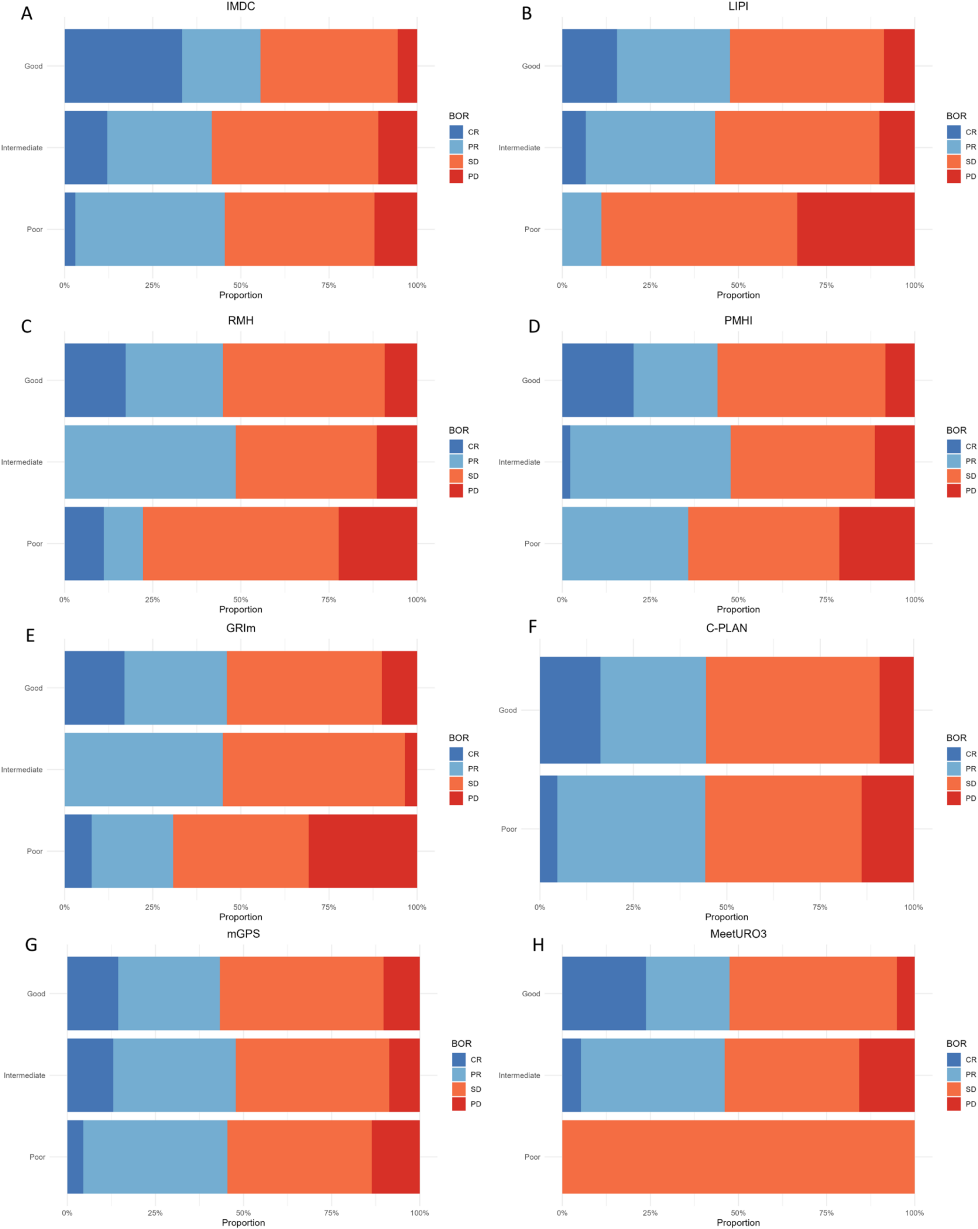
Distribution of best overall responses (BOR) by risk groups of each prognostic score (A: IMDC, B: LIPI, C: RMH, D: PMHI, E: GRIm, F: C‐PLAN, G: mGPS, H: Meet‐URO3). The BOR distribution was calculated for each risk group within each prognostic score. The distribution is displayed using the following colors: CR (blue), PR (light blue), SD (orange), and PD (red).

We examined the association of each score with treatment response, assessing how each score was related to ORR and PD. Figure 2A–H shows the percentage of best treatment effects for each risk. The best overall response (BOR) ranges for each risk group were: CR 14.4%–33.3%, PR 22.2%–32.0%, SD 38.9%–47.6%, and PD 5.1%–10.3% in the Good group; CR 0%–13.0%, PR 29.7%–48.6%, SD 38.2%–51.7%, and PD 3.4%–15.8% in the Intermediate group; and CR 0%–11.1%, PR 0%–42.4%, SD 38.5%–100%, and PD 0%–33.3% in the Poor group. Although there was an overall bias in the number of cases by risk group, there was a tendency toward decreased CR and increased PD in the Poor group. The Good group showed higher rates of CR and PR while the Poor group had increased PD and SD; however, the Intermediate group was inconsistent in Cochran‐Armitage trends and the distribution differed for each score. Although the decrease in CR associated with increased risk was significant for some scores (minimum *p*‐value = 0.002), the increase in PD was not significant for all scores.

**FIGURE 3:**
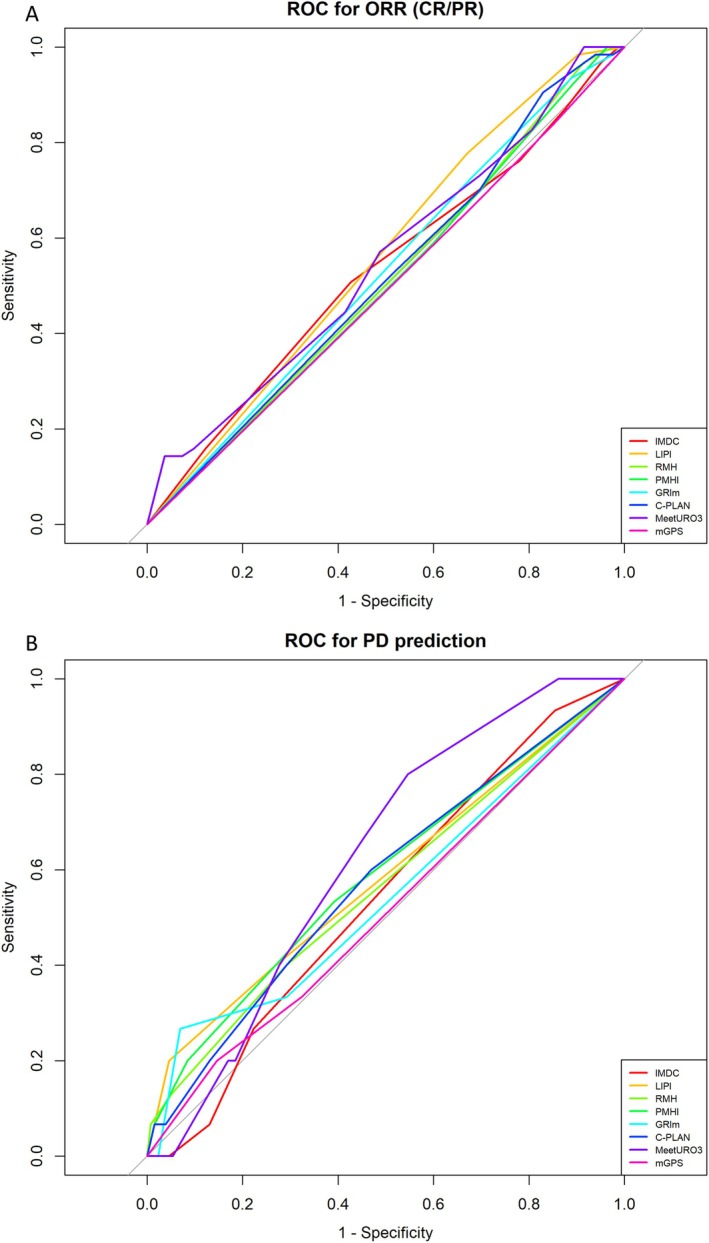
ROC curve for PD and ORR by each prognostic score (A: PD, B: ORR). ROC curves for PD and ORR (CR + PR) were plotted using the point of each prognostic score as a factor. Prognostic scores are displayed as follows: IMDC (red), LIPI (orange), RMH (green), PMHI (light green), GRIm (light blue), C‐PLAN (blue), Meet‐URO3 (purple), and mGPS (pink).

A ROC analysis was then performed for PD and ORR. The ROC curve was linear because of few variables, and there were no significant differences observed between the scores (Figure 3A,B). The AUC ranged from 0.513 (mGPS) to 0.627 (Meet‐URO3) for PD and from 0.491 (mGPS) to 0.562 (LIPI) for ORR (Table 3). No scores showed discriminative ability for treatment response.

**TABLE 3:** AUC for predicting PD and ORR by each prognostic score.

Score	AUC outcome	
	PD	ORR
IMDC	0.541	0.531
LIPI	0.583	0.562
RMH	0.564	0.510
PMHI	0.588	0.503
GRIm	0.545	0.528
C‐PLAN	0.576	0.517
mGPS	0.513	0.491
Meet‐URO3	0.627	0.550

Abbreviations: AUC: area under the curve; GRIm: Gustave Roussy Immune Score; IMDC: International Metastatic RCC Database Consortium; LIPI: Lung Immune Prognostic Index; mGPS: modified Glasgow Prognostic Score; ORR: objective response rate; PD: progressive disease; PMHI: Princess Margaret Hospital Index; RMH: Royal Marsden Hospital score.

### 3.4 Correlation of Prognostic Scores With PFS and OS

**FIGURE 4:**
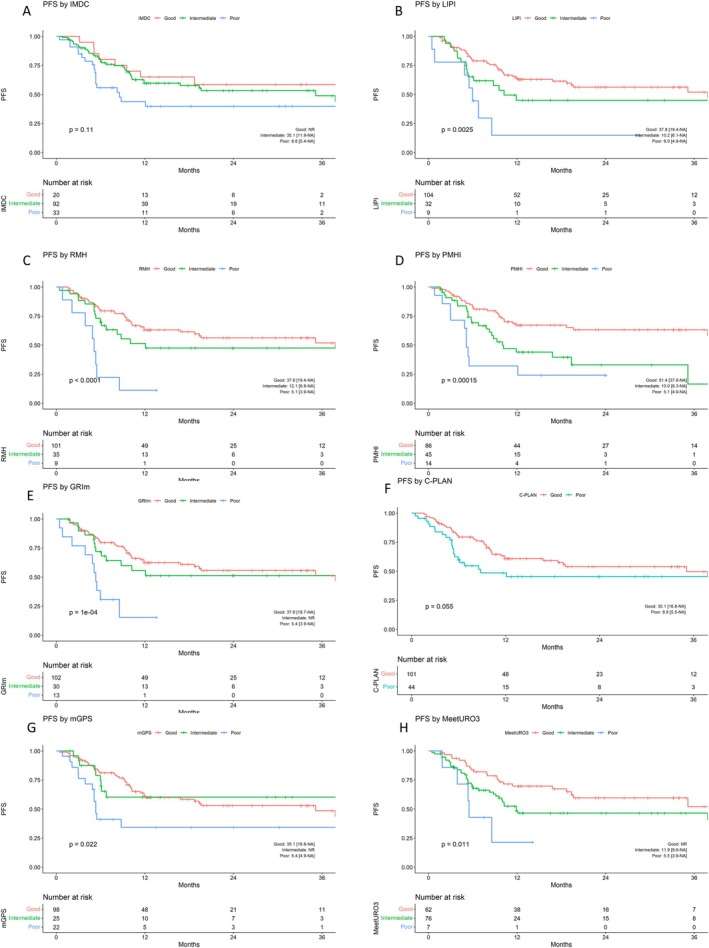
Kaplan–Meier curves for PFS by each prognostic score (A: IMDC, B: LIPI, C: RMH, D: PMHI, E: GRIm, F: C‐PLAN, G: mGPS, H: Meet‐URO3). PFS was stratified by risk group for each prognostic score and plotted using the Kaplan–Meier method. The risk groups are displayed as follows: Good (red), Intermediate (green), and Poor (blue).

**FIGURE 5:**
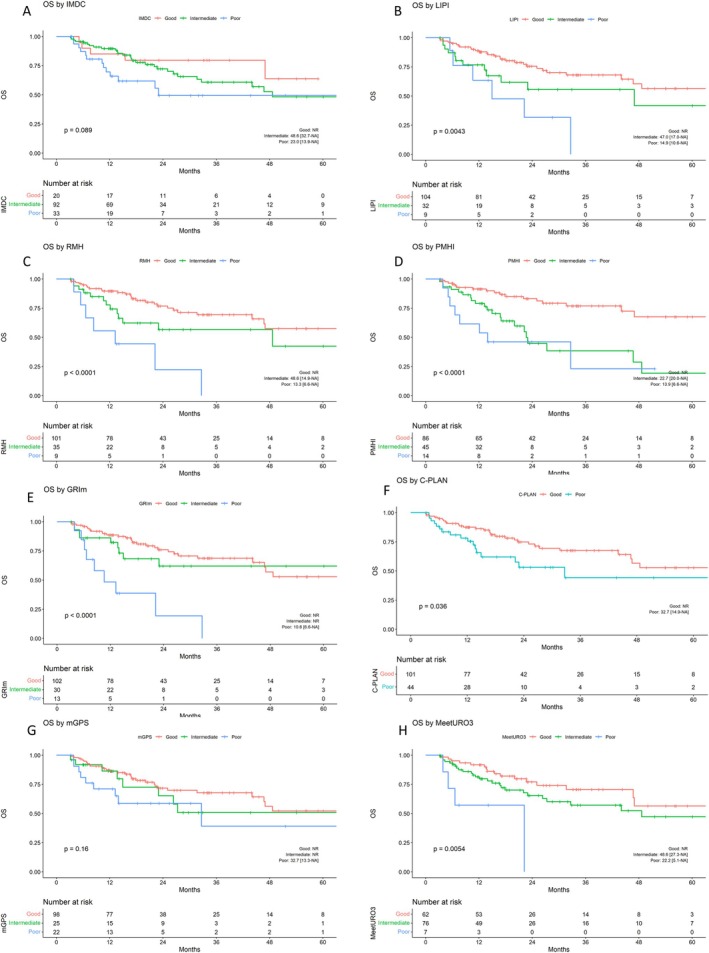
Kaplan–Meier curves for OS by each prognostic score (A: IMDC, B: LIPI, C: RMH, D: PMHI, E: GRIm, F: C‐PLAN, G: mGPS, H: Meet‐URO3). OS was stratified by risk group for each prognostic score and plotted using the Kaplan–Meier method. The risk groups are displayed as follows: Good (red), Intermediate (green), and Poor (blue).

**FIGURE 6:**
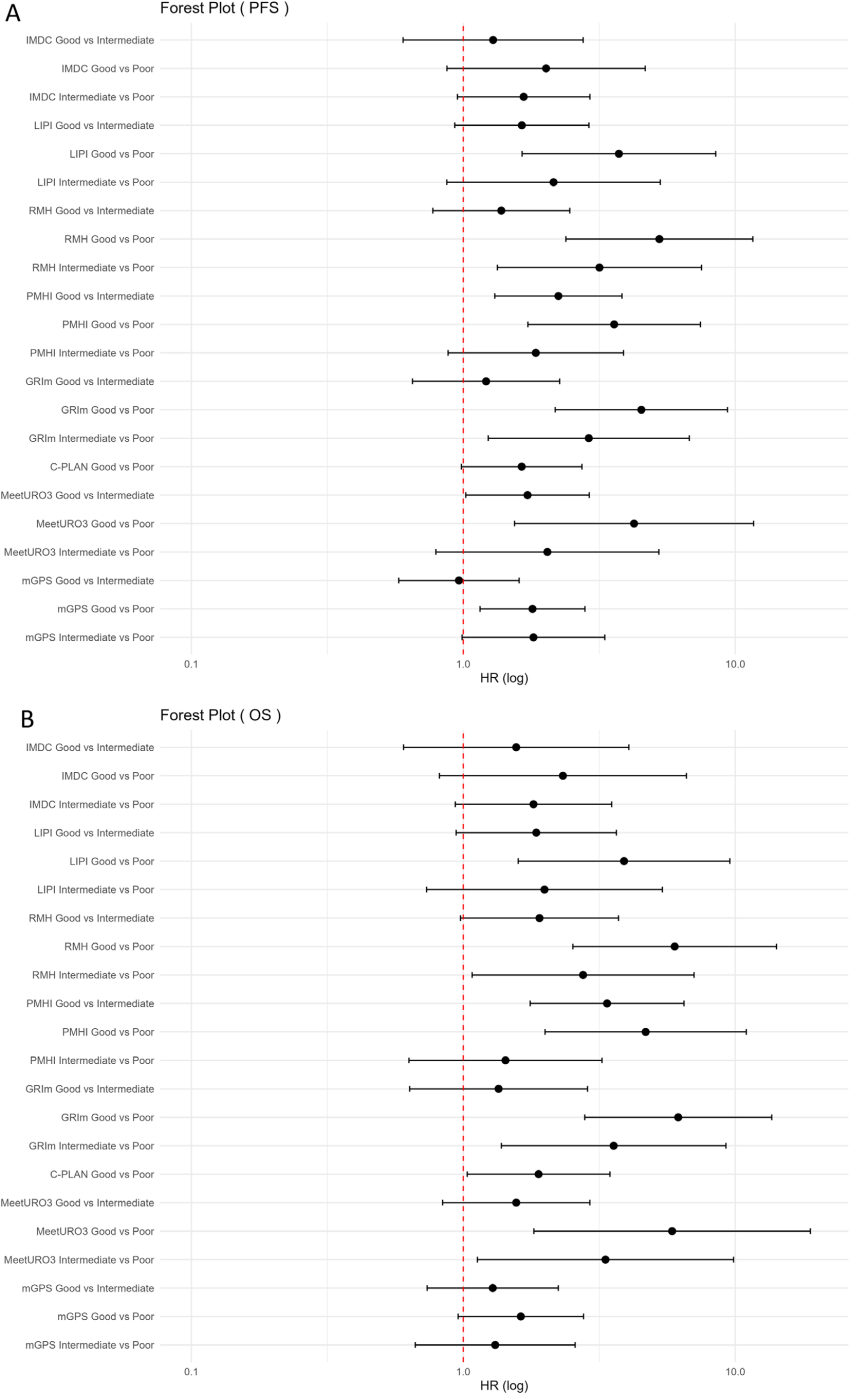
Cox regression analysis between risk groups for each prognostic score (A: PFS, B: OS). For each prognostic score, the hazard ratios between two risk groups are evaluated using Cox proportional hazards models for the following comparisons: Good versus Intermediate, Good versus Poor, and Intermediate versus Poor.

The median follow‐up was 28.8 months, with a median PFS of 35.1 months, and the median OS was not reached. Survival analysis of each score was performed by Kaplan–Meier analysis in order to compare the prognostic efficacy in PFS and OS (Figures 4A–H and 5A–H). Although almost all Kaplan–Meier curves showed good visual separation between each risk group, there were no significant differences between IMDC or C‐PLAN for PFS and IMDC or mGPS for OS. Next, the Cox regression analysis was used to identify significant differences between risk groups. All scores that showed significant differences in overall log‐rank test were significantly poorer in the Good versus Poor group (PFS HR 1.80–5.26, OS HR 1.89–6.17). Furthermore, Intermediate versus Poor with regard to RMH (PFS HR 3.17, *p*‐value = 0.009; OS HR 2.76, *p*‐value = 0.034) and GRIm (PFS HR 2.89, *p*‐value = 0.014; OS HR 3.57, *p*‐value = 0.009), plus Good versus Intermediate with regard to PMHI (PFS HR 2.24, *p*‐value = 0.003; OS HR 3.38, *p*‐value < 0.001) and Meet‐URO3 (PFS HR 1.72, *p*‐value = 0.041), were significantly associated with poor prognoses (Figure 6A,B). Additionally, multivariate Cox regression analyses, adjusted for age, sex, and treatment regimen (ICI‐ICI vs. ICI‐TKI), were performed to evaluate whether each prognostic score had prognostic ability independent of confounding factors. The results of the adjusted Cox regression analysis (Tables S2 and S3) were consistent with main Cox regression analyses. In particular, PMHI was the only score to show a significant difference between the intermediate group and the good group for both PFS and OS.

The C‐index results are summarized in Table 4. The top three scores for PFS were PMHI (0.708), RMH (0.683), and GRIm (0.678) while those for OS were PMHI (0.755), RMH (0.728), and GRIm (0.726). Collectively, the PMHI showed a C‐index over 0.7 for PFS and OS, with PMHI being the most useful prognostic score for cancer immunotherapy. Additionally, to compare outcomes by treatment regimen, the C‐index was evaluated separately for the ICI‐ICI group and the ICI‐TKI group (Table S4). The C‐index for each score was generally similar between the two treatment groups. Furthermore, subgroup analyses were performed within IMDC‐defined risk categories. The C‐index for each score regarding PFS and OS was evaluated in the IMDC intermediate‐ and poor‐risk groups (Table S5). Notably, while mGPS showed the highest C‐index in the intermediate‐risk group, no single score demonstrated the highest performance consistently in the high‐risk group. Time‐dependent ROC analyses for PFS and OS at 12, 36, and 60 months are shown in Figure S1 and Table S6. These results were consistent with those of the C‐index. In particular, PMHI (PFS at 36 months, AUC 0.756; OS at 36 months, AUC 0.723; OS at 60 months, AUC 0.715) and Meet‐URO3 (OS at 12 months, AUC 0.715) showed good performance.

**TABLE 4:** C‐index for PFS and OS for each prognostic score.

Score	C‐index	
	PFS	OS
IMDC	0.629	0.643
LIPI	0.675	0.700
RMH	0.683	0.728
PMHI	0.708	0.755
GRIm	0.678	0.726
C‐PLAN	0.654	0.686
mGPS	0.659	0.652
Meet‐URO3	0.636	0.661

Abbreviations: GRIm: Gustave Roussy Immune Score; IMDC: International Metastatic RCC Database Consortium; LIPI: Lung Immune Prognostic Index; mGPS: modified Glasgow Prognostic Score; OS: overall survival; PFS: progression‑free survival; PMHI: Princess Margaret Hospital Index; RMH: Royal Marsden Hospital score.

## 4 Discussion

In this study, we evaluated the performance of several prognostic scores for combination immunotherapy using first‐line ICI for mRCC. Kaplan–Meier analysis showed significant differences in all scores, except between IMDC and C‐PLAN in PFS and between IMDC and mGPS in OS, confirming at least partial prognostic ability for prognostic scoring in mRCC. In the Cox regression analysis, the difference was especially significant in the Good vs. Intermediate group with regard to the PMHI, suggesting enhanced sensitivity versus other scores. In the C‐index, PMHI also showed the highest discriminative ability for both PFS and OS. Overall, most scores showed at least partial ability for predicting PFS and OS, with PMHI being the most useful. In contrast, no score accurately predicted PD or ORR.

Previous reports examining prognostic scores for various cancer types found that LIPI is most strongly associated with PD, OS, and PFS during ICI treatment [4]. LIPI was first reported to have prognostic ability for ICI treatment of melanoma [11], a finding bolstered by subsequent studies [20, 21]. In urological cancers, several reports have focused on metastatic urothelial carcinoma [22] and renal cell carcinoma [23], showing that pretreatment LIPI correlated with worse survival outcomes in mRCC patients receiving ICI or TKI therapy. In particular, the findings regarding mRCC suggest that LIPI may function as a prognostic stratification marker reflecting inflammatory condition and tumor burden in mRCC rather than as an indicator for any specific treatment regimen. This interpretation is consistent with reports in other solid tumors. Thus, a growing body of evidence indicates that LIPI may be an excellent prognostic score for immunotherapy, regardless of cancer type. However, while most reports evaluate PFS and OS, very few reports mention associations with treatment response parameters (such as ORR) [21, 24]. In the present study, while LIPI demonstrated prognostic ability for PFS and OS, it could not do the same for treatment efficacy. This may explain why such results are underreported. As LIPI is based on the derived neutrophil‐to‐lymphocyte ratio (dNLR) and lactate dehydrogenase levels, it indexes inflammation with tissue injury and has a fairly robust applicability to any solid cancer type [21]. However, mRCC features aggressive metastasis, which is a parameter difficult to quantify with LIPI in later disease stages.

In addition to LIPI, evidence regarding some prognostic scores in mRCC is accumulating within the literature. Recently, several mRCC studies have examined mGPS and the Meet‐URO score since mGPS, which is based on CRP and albumin, is an independent prognostic factor in mRCC and its prognostic ability has been shown equivalent to or superior to IMDC [25, 26]. Furthermore, the Meet‐URO score, which adds NLR and bone metastases to the IMDC, showed superior prognostic stratification compared to IMDC in patients treated with nivolumab, nivolumab plus ipilimumab, or pembrolizumab plus axitinib [8, 17]. Its prognostic accuracy has also been confirmed in older patients (≥ 70 years) treated with cabozantinib or nivolumab‐based immunotherapy [27, 28]. This score generally classifies into five risk groups; however, some studies have reported modified three‐group classification [18, 19]. In cases other than first‐line treatment of mRCC, there are reports indicating that RMH correlates with OS and PFS in rare cancers, such as digestive system and female genital tract cancers [29], as well as in meta‐analyses of multiple cancer types (including colorectal and lung cancers) [30]. RMH and GRIm are also reported useful for predicting prognosis in ICI treatment cases with prior treatment history, specifically in non‐small‐cell lung cancer [31] and small‐cell lung cancer [32]. Likewise, reports on C‐PLAN are limited although it has been reported in cases of first‐line ICI therapy for non‐small‐cell lung cancer [15] and second‐line or later nivolumab treatment for mRCC [33]. While several reports judged C‐PLAN as useful for predicting PFS and OS, its prognostic performance remains limited.

In the present study, a cross‐sectional comparison of prognostic scores for PFS and OS using C‐index analyses revealed that PMHI was the best performer. Originally developed over multiple phase I trials for molecularly targeted agents against advanced solid cancers to predict early death within 90 days at the Princess Margaret Hospital [13], PMHI reflects nutritional status, tumor burden, and general condition. These factors are based on poor prognostic factors common to patients with solid tumors (e.g., gastrointestinal, thoracic/head and neck, breast/gynecological cancers, and/or urologic cancers) and may be a valid prognostic score for mRCC. The RMH score, a potential competitor, also reflects nutritional status and tumor burden but the reason for the better performance of PMHI over RMH may be the inclusion of PS as an indicator reflective of systemic status. The IMDC risk classification, which is widely used in the management of renal cell carcinoma, uses the Karnofsky Performance Status (KPS) score, with a KPS < 80% considered equivalent to PS ≥ 1. Therefore, PMHI may have higher resolution regarding systemic status.

Interestingly, in this study, prognostic scores that incorporated inflammatory markers, such as CRP, NLR, and dNLR, were inferior to those that excluded them, such as PMHI and RMH. In spite of the good performance of LIPI, we believe that, unlike tumor burden and systemic conditions (which reflect long‐term and constant disease states), inflammatory markers reflect short‐term and temporary disease states. In fact, inflammatory markers may vary due to factors unrelated to tumors (such as infection), and both general condition and solid tumor status may be influenced by inflammation; this was seen in several reports where early inflammatory kinetics were strongly associated with prognoses [34, 35]. Therefore, for mRCC, it has been suggested that scores inclusive of long‐term and constant disease states, such as tumor burden and systemic status, may be preferable when combining multiple biomarkers.

ROC analysis revealed no useful scores for either PD or ORR. This result is similar to previous reports showing that prognostic scores only predict PFS and OS and have low prognostic ability for treatment efficacy [11, 31]. However, the evaluation of prediction accuracy by ROC analysis is greatly affected by factors such as variable continuity. Since the prognostic scores examined in this study had a limited number of component variables, ranging from two for LIPI and mGPS to six for IMDC, this restricted number of variables may have reduced the resolution of the ROC analysis. Therefore, it should be noted that the accuracy of ROC analysis for prognostic scores is inherently limited.

Based on these findings, the evaluated scores should be interpreted primarily as pretreatment prognostic tools rather than as indicators of treatment response. In clinical settings, scores such as the PMHI and RMH can contribute to supporting baseline risk stratification, estimating survival prognosis, and providing patient counseling before initiating first‐line treatment for mRCC. However, these scores should not be used alone to determine a treatment regimen or predict a short‐term treatment response.

The limitations of this study are the retrospective design and small number of cases. Additionally, this database did not include molecular biomarkers or tumor microenvironment‐related data which prevented integrated novel biomarker analyses or nomogram construction. Moreover, the cutoff values used for scoring may differ from those previously reported for Japanese populations. Furthermore, as the analysis combined both the ICI‐ICI and ICI‐TKI groups, there was some risk of treatment selection bias. Additionally, the distribution of prognostic groups across the evaluated scores was imbalanced since the good and intermediate risk groups accounted for the majority of cases while the poor risk group remained in the minority. This imbalance, along with the fact that C‐PLAN is a two‐group classification, may have affected cross‐validation comparisons of the scores. However, to our best knowledge, few studies have cross‐sectionally compared multiple prognostic scores in patients with mRCC receiving first‐line combination immunotherapy, and our findings suggest that PMHI may have prognostic ability in this setting. Further studies integrating clinical scores with molecular biomarkers and tumor microenvironment‐related data are needed to develop more accurate prognostic models.

## 5 Conclusion

Here, PMHI and RMH showed the highest prognostic performance among the evaluated clinical scores as pretreatment prognostic tools before the start of combination immunotherapy in patients with mRCC. As both can be easily calculated during daily practice with common clinical data collected at diagnosis, we consider that they may be useful for baseline risk stratification, estimation of survival outcomes, and patient counseling, especially PMHI, because they reflect long‐term disease and systemic status. However, their ability to predict treatment response, including ORR and PD, was limited.

## Author Contributions

**Akifumi Omiya:** data curation, resources, investigation. **Shuya Kandori:** writing – review and editing, methodology, supervision, resources, data curation, conceptualization. **Hiromitsu Negoro:** resources. **Manabu Komine:** resources. **Shota Takahashi:** data curation, investigation, resources. **Kotoe Matsuda:** data curation, investigation, resources. **Hiromichi Sakurai:** writing – original draft, methodology, conceptualization, software, formal analysis, data curation, visualization, writing – review and editing, investigation. **Tomoki Ishida:** data curation, investigation, resources. **Masanobu Shiga:** supervision, resources, data curation, methodology, writing – review and editing. **Bunpei Isoda:** data curation, investigation, resources. **Bryan J. Mathis:** writing – review and editing. **Takamitsu Inoue:** resources. **Jun Miyazaki:** resources.

## Funding

This study was supported by Co‐Creation Place Formation Support Program COI‐NEXT (no. JPMJPF2017).

## Ethics Statement

This study was approved by the Ethics Committee of the University of Tsukuba Hospital (Approval No. R05‐057, August 25, 2023).

## Conflicts of Interest

Shuya Kandori received honoraria from Bristol Myers Squibb. Hiroyuki Nishiyama reports grants and personal fees from Astellas, grants from Chugai, and personal fees from Bristol Myers Squibb, Janssen, MSD, Ono, Pfizer, AstraZeneca, Nippon Kayaku Co. Ltd., and Merck Biopharma Co. Ltd. outside the submitted work. The other authors declare no conflicts of interest. The funding source had no role in the design, practice, or analysis of this study.

## Supporting information


**Figure S1:** Time‐dependent receiver operating characteristic analyses for progression‐free survival and overall survival at 12, 36, and 60 months. Curves are shown for each prognostic score as follows: IMDC (red), LIPI (orange), RMH (green), PMHI (light green), GRIm (light blue), C‐PLAN (blue), Meet‐URO3 (purple), and mGPS (pink).

**Figure S2:** Patient selection flow chart. Of 165 consecutive patients with histologically diagnosed metastatic renal cell carcinoma who started first‐line combination immunotherapy, 20 patients with missing data required for prognostic score calculation were excluded, and 145 patients were included in the final analysis.

**Table S1:** Patient characteristics at the start of first‐line combination immunotherapy in the ICI‐ICI and ICI‐TKI groups.
**Table S2:** Multivariable Cox regression analysis for progression‐free survival, adjusted for age, sex, and treatment regimen.
**Table S3:** Multivariable Cox regression analysis for overall survival, adjusted for age, sex, and treatment regimen.
**Table S4:** C‐index for progression‐free survival and overall survival by treatment regimen.
**Table S5:** Subgroup analysis of C‐index for progression‐free survival and overall survival in the IMDC intermediate‐ and poor‐risk groups.
**Table S6:** Time‐dependent AUC values for progression‐free survival and overall survival at 12, 36, and 60 months.

## Data Availability

The data that support the findings of this study are available from the corresponding author upon reasonable request.
