# Supplementary figures and images for: Assessment of Multiple Prognostic Scores in Patients With Metastatic Renal Cell Carcinoma Receiving First‐Line, Immune‐Based Combinations
Source: Cancer Rep (Hoboken). 2026 Jun 16;9(6):e70604. doi: 10.1002/cnr2.70604 (PMC13272634; doi:10.1002/cnr2.70604)

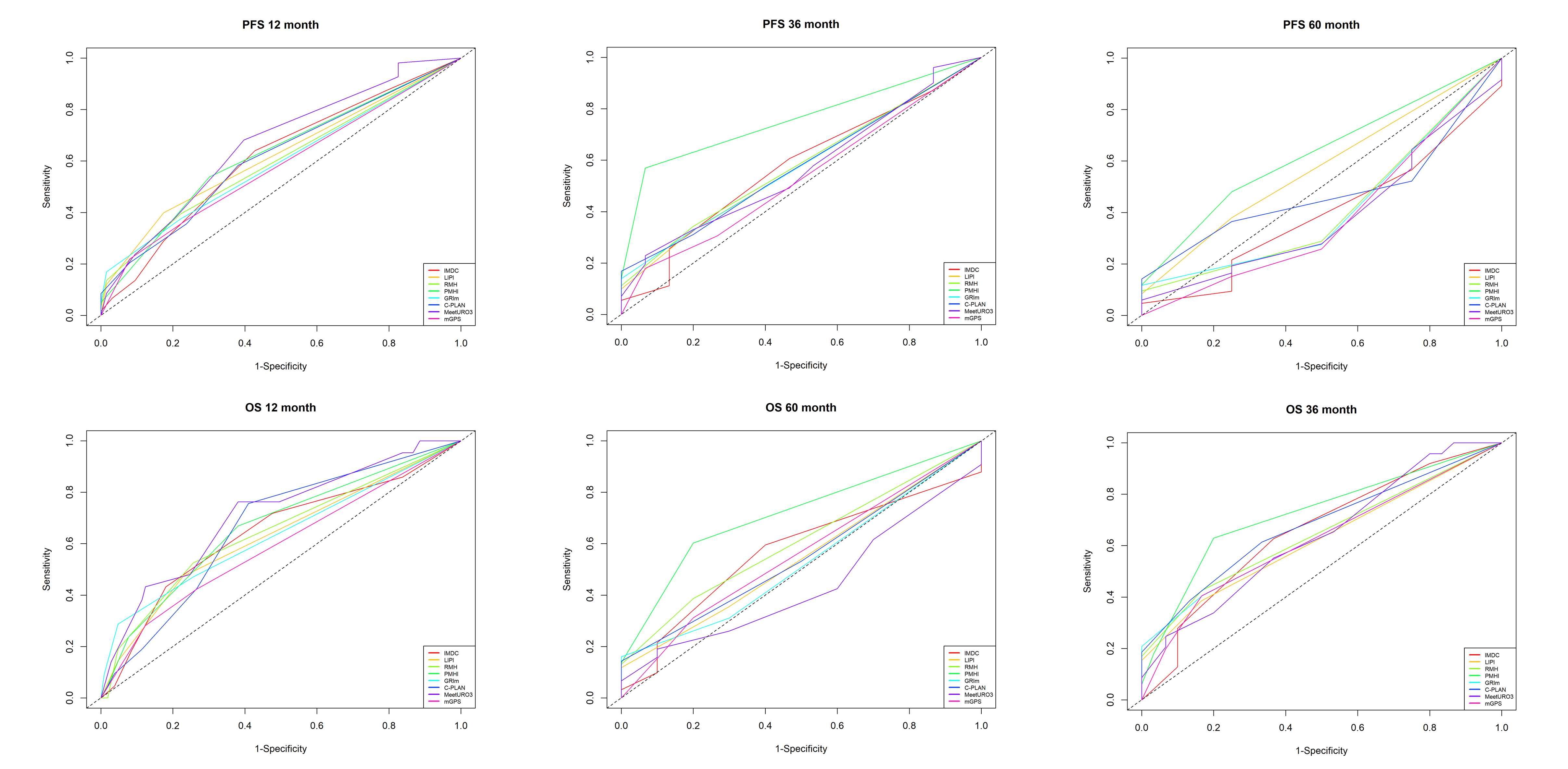

Supplement: Supplementary file 1 — Figure S1: Time‐dependent receiver operating characteristic analyses for progression‐free survival and overall survival at 12, 36, and 60 months. Curves are shown for each prognostic score as follows: IMDC (red), LIPI (orange), RMH (green), PMHI (light green), GRIm (light blue), C‐PLAN (blue), Meet‐URO3 (purple), and mGPS (pink). [file CNR2-9-e70604-s002.tif]

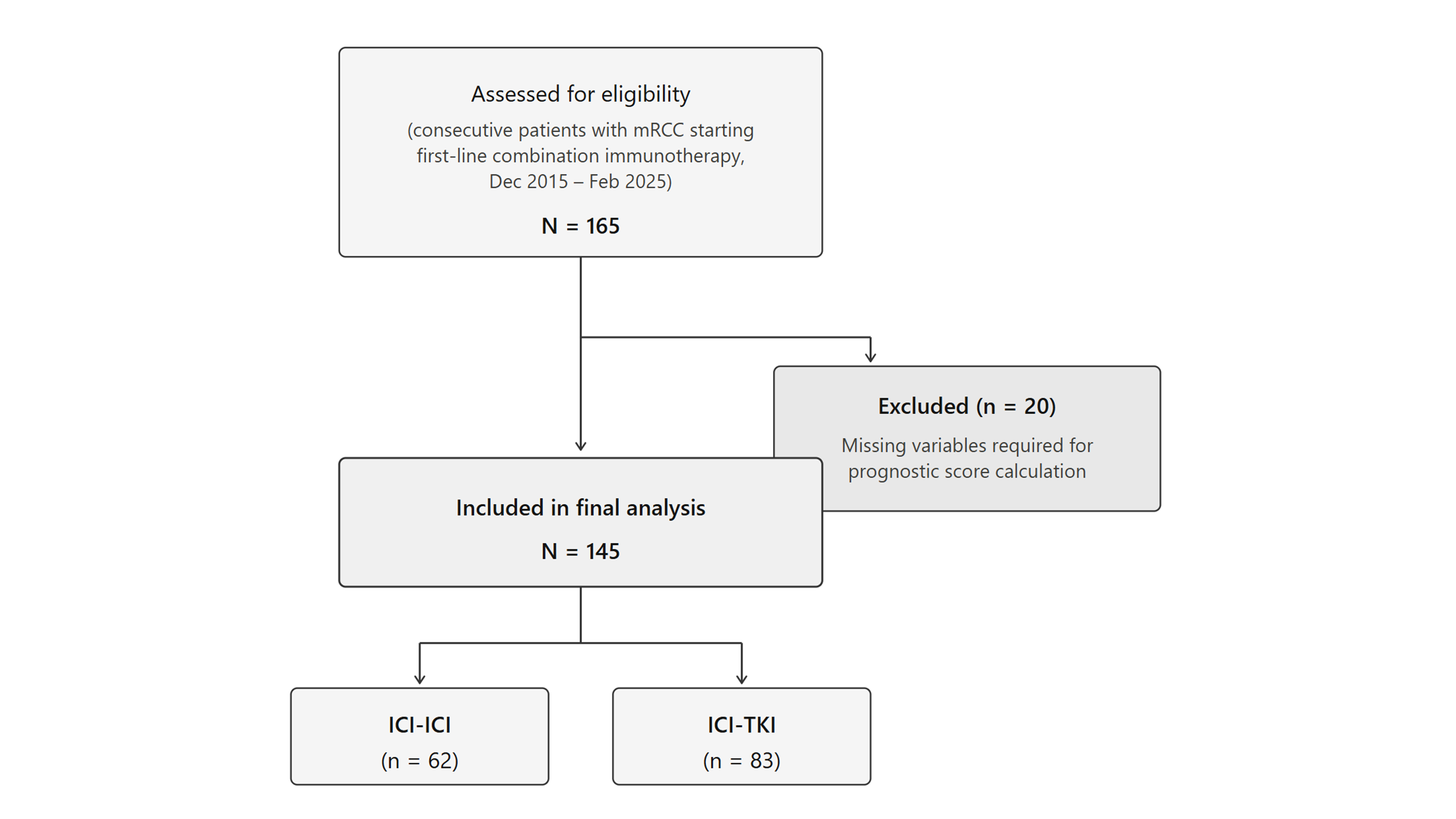

Supplement: Supplementary file 2 — Figure S2: Patient selection flow chart. Of 165 consecutive patients with histologically diagnosed metastatic renal cell carcinoma who started first‐line combination immunotherapy, 20 patients with missing data required for prognostic score calculation were excluded, and 145 patients were included in the final analysis. [file CNR2-9-e70604-s003.tif]
