# Supplementary material for: Assessment of Multiple Prognostic Scores in Patients With Metastatic Renal Cell Carcinoma Receiving First‐Line, Immune‐Based Combinations
Source: Cancer Rep (Hoboken). 2026 Jun 16;9(6):e70604. doi: 10.1002/cnr2.70604 (PMC13272634; doi:10.1002/cnr2.70604)
Supplement: Supplementary file 3 — Table S1: Patient characteristics at the start of first‐line combination immunotherapy in the ICI‐ICI and ICI‐TKI groups. Table S2: Multivariable Cox regression analysis for progression‐free survival, adjusted for age, sex, and treatment regimen. Table S3: Multivariable Cox regression analysis for overall survival, adjusted for age, sex, and treatment regimen. Table S4: C‐index for progression‐free survival and overall survival by treatment regimen. Table S5: Subgroup analysis of C‐index for progression‐free survival and overall survival in the IMDC intermediate‐ and poor‐risk groups. Table S6: Time‐dependent AUC values for progression‐free survival and overall survival at 12, 36, and 60 months. [file CNR2-9-e70604-s001.docx]

Supplementary Table S1. Patient characteristics at the start of first-line combination immunotherapy in the ICI-ICI vs. ICI-TKI groups

|  | ICI-ICI (n = 62) | ICI-TKI (n = 83) | P-value |
| --- | --- | --- | --- |
| Age at Treatment Initiation  Median [IQR] | 68 [61-74] | 71 [65-77] | 0.035 |
| Sex |  |  | 1 |
| Male | 50 (80.6%) | 67 (80.7%) |  |
| Female | 12 (19.4%) | 16 (19.3%) |  |
| PS (Performance Status) |  |  | 0.3 |
| 0 | 47 (75.8%) | 58 (69.9%) |  |
| ≥1 | 15 (24.2%) | 25 (30.1%) |  |
| IMDC risk |  |  | 0.003 |
| Favorable | 2 (3.2%) | 18 (21.7%) |  |
| Intermediate | 45 (72.6%) | 47 (56.6%) |  |
| Poor | 15 (24.2%) | 18 (21.7%) |  |
| Pathology |  |  | 0.802 |
| Clear | 54 (87.1%) | 70 (84.3%) |  |
| Others | 8 (12.9%) | 13 (15.7%) |  |
| Timing of Metastasis |  |  | 0.616 |
| Synchronous | 35 (56.5%) | 43 (51.8%) |  |
| Metachronous | 27 (43.5%) | 40 (48.2%) |  |
| Metastatic Site |  |  |  |
| Lung | 42 (67.7%) | 54 (65.1%) | 0.859 |
| Brain | 2 (3.2%) | 3 (3.6%) | 1 |
| Liver | 4 (6.5%) | 6 (7.2%) | 1 |
| Bone | 16 (25.8%) | 21 (25.3%) | 1 |
| Lymph node | 21 (33.9%) | 23 (27.7%) | 0.468 |

IQR: Interquartile Range; PS: performance status; IMDC: International Metastatic Renal Cell Carcinoma Database Consortium

Supplementary Table S2. Multivariable Cox regression analysis for PFS, adjusted for age, sex, and treatment regimen

| Score | Comparison | HR | 95% CI | p-value |
| --- | --- | --- | --- | --- |
| IMDC | Favorable (ref) | ― | ― | ― |
|  | Intermediate vs Good | 1.1 | 0.48-2.50 | 0.82 |
|  | Poor vs Good | 1.97 | 0.83-4.68 | 0.12 |
| LIPI | Good (ref) | ― | ― | ― |
|  | Intermediate vs Good | 1.75 | 0.98-3.11 | 0.06 |
|  | Poor vs Good | 3.85 | 1.69-8.79 | 0.00 |
| RMH | Good (ref) | ― | ― | ― |
|  | Intermediate vs Good | 1.4 | 0.78-2.50 | 0.26 |
|  | Poor vs Good | 5.24 | 2.38-11.55 | <0.01 |
| PMHI | Good (ref) | ― | ― | ― |
|  | Intermediate vs Good | 2.18 | 1.27-3.75 | <0.01 |
|  | Poor vs Good | 4.09 | 1.96-8.53 | <0.01 |
| GRIm | Good (ref) | ― | ― | ― |
|  | Intermediate vs Good | 1.26 | 0.67-2.38 | 0.47 |
|  | Poor vs Good | 4.47 | 2.15-9.26 | <0.01 |
| C-PLAN | Poor vs Good | 1.69 | 1.01-2.82 | 0.05 |
| mGPS | Good (ref) | ― | ― | ― |
|  | Intermediate vs Good | 0.97 | 0.47-2.00 | 0.93 |
|  | Poor vs Good | 2.36 | 1.25-4.45 | 0.01 |
| Meet-URO3 | Good (ref) | ― | ― | ― |
|  | Intermediate vs Good | 1.7 | 1.00-2.88 | 0.05 |
|  | Poor vs Good | 3.75 | 1.39-10.09 | 0.01 |

PFS: progression-free survival; IMDC: International Metastatic RCC Database Consortium; LIPI: Lung Immune Prognostic Index; RMH: Royal Marsden Hospital score; PMHI: Princess Margaret Hospital Index; GRIm: Gustave Roussy Immune Score; mGPS: modified Glasgow Prognostic Score

Supplementary Table S3. Multivariable Cox regression analysis for OS, adjusted for age, sex, and treatment regimen

| Score | Comparison | HR | 95% CI | p-value |
| --- | --- | --- | --- | --- |
| IMDC | Favorable (ref) | ― | ― | ― |
|  | Intermediate vs Good | 1.23 | 0.44-3.47 | 0.69 |
|  | Poor vs Good | 2.68 | 0.90-7.98 | 0.08 |
| LIPI | Good (ref) | ― | ― | ― |
|  | Intermediate vs Good | 2.17 | 1.07-4.40 | 0.03 |
|  | Poor vs Good | 3.96 | 1.61-9.73 | <0.01 |
| RMH | Good (ref) | ― | ― | ― |
|  | Intermediate vs Good | 1.91 | 0.98-3.76 | 0.06 |
|  | Poor vs Good | 5.81 | 2.45-13.79 | <0.01 |
| PMHI | Good (ref) | ― | ― | ― |
|  | Intermediate vs Good | 3.07 | 1.58-5.96 | <0.01 |
|  | Poor vs Good | 4.66 | 2.00-10.86 | <0.01 |
| GRIm | Good (ref) | ― | ― | ― |
|  | Intermediate vs Good | 1.46 | 0.67-3.17 | 0.34 |
|  | Poor vs Good | 5.6 | 2.53-12.38 | <0.01 |
| C-PLAN | Poor vs Good | 1.96 | 1.06-3.61 | 0.03 |
| mGPS | Good (ref) | ― | ― | ― |
|  | Intermediate vs Good | 1.44 | 0.65-3.19 | 0.37 |
|  | Poor vs Good | 1.98 | 0.93-4.22 | 0.08 |
| Meet-URO3 | Good (ref) | ― | ― | ― |
|  | Intermediate vs Good | 1.51 | 0.80-2.84 | 0.20 |
|  | Poor vs Good | 5.98 | 1.91-18.74 | <0.01 |

OS: overall survival; IMDC: International Metastatic RCC Database Consortium; LIPI: Lung Immune Prognostic Index; RMH: Royal Marsden Hospital score; PMHI: Princess Margaret Hospital Index; GRIm: Gustave Roussy Immune Score; mGPS: modified Glasgow Prognostic Score.

Supplementary Table S4. C-index for PFS and OS by treatment regimen

|  | PFS | |  | OS | |
| --- | --- | --- | --- | --- | --- |
| Score | ICI-ICI | ICI-TKI |  | ICI-ICI | ICI-TKI |
| IMDC | 0.634 (0.518-0.751) | 0.636 (0.510-0.763) |  | 0.669 (0.540-0.797) | 0.632 (0.455-0.810) |
| LIPI | 0.670 (0.507-0.834) | 0.700 (0.565-0.836) |  | 0.694 (0.547-0.841) | 0.736 (0.572-0.900) |
| RMH | 0.698 (0.551-0.845) | 0.678 (0.539-0.817) |  | 0.709 (0.568-0.850) | 0.754 (0.602-0.907) |
| PMHI | 0.777 (0.655-0.898) | 0.655 (0.515-0.795) |  | 0.736 (0.593-0.880) | 0.788 (0.655-0.920) |
| GRIm | 0.712 (0.565-0.859) | 0.662 (0.524-0.799) |  | 0.715 (0.566-0.863) | 0.751 (0.605-0.898) |
| C-PLAN | 0.669 (0.536-0.801) | 0.651 (0.538-0.765) |  | 0.664 (0.539-0.790) | 0.720 (0.613-0.827) |
| mGPS | 0.669 (0.505-0.833) | 0.652 (0.500-0.805) |  | 0.660 (0.484-0.835) | 0.639 (0.444-0.835) |
| Meet-URO3 | 0.608 (0.493-0.723) | 0.659 (0.550-0.767) |  | 0.640 (0.517-0.764) | 0.687 (0.543-0.831) |

C-index: Harrell's concordance index; PFS: progression-free survival; OS: overall survival; IMDC: International Metastatic RCC Database Consortium; LIPI: Lung Immune Prognostic Index; RMH: Royal Marsden Hospital score; PMHI: Princess Margaret Hospital Index; GRIm: Gustave Roussy Immune Score; mGPS: modified Glasgow Prognostic Score.

Supplementary Table S5. Subgroup analysis of C-index for PFS and OS in the IMDC intermediate- and poor-risk groups

|  | PFS | |  | OS | |
| --- | --- | --- | --- | --- | --- |
| Score | Intermediate | Poor |  | Intermediate | Poor |
| LIPI | 0.664 (0.512-0.817) | 0.622 (0.442-0.802) |  | 0.745 (0.598-0.892) | 0.576 (0.366-0.787) |
| RMH | 0.624 (0.433-0.814) | 0.609 (0.431-0.787) |  | 0.703 (0.527-0.880) | 0.646 (0.431-0.860) |
| PMHI | 0.661 (0.512-0.810) | 0.673 (0.487-0.858) |  | 0.724 (0.579-0.869) | 0.678 (0.487-0.869) |
| GRIm | 0.623 (0.438-0.807) | 0.622 (0.455-0.788) |  | 0.719 (0.535-0.903) | 0.617 (0.427-0.808) |
| C-PLAN | 0.668 (0.537-0.799) | 0.498 (0.348-0.649) |  | 0.704 (0.570-0.838) | 0.452 (0.243-0.661) |
| mGPS | 0.766 (0.632-0.900) | 0.464 (0.265-0.663) |  | 0.786 (0.658-0.915) | 0.412 (0.180-0.644) |
| Meet-URO3 | 0.604 (0.482-0.727) | 0.602 (0.419-0.784) |  | 0.601 (0.446-0.756) | 0.705 (0.554-0.857) |

IMDC intermediate-risk group: n = 92; IMDC poor-risk group: n = 33.

C-index: Harrell's concordance index; PFS: progression-free survival; OS: overall survival; IMDC: International Metastatic RCC Database Consortium; LIPI: Lung Immune Prognostic Index; RMH: Royal Marsden Hospital score; PMHI: Princess Margaret Hospital Index; GRIm: Gustave Roussy Immune Score; mGPS: modified Glasgow Prognostic Score.

Supplementary Table S6. Time-dependent AUC for PFS and OS by each prognostic score

| Score | PFS (AUC) | | | OS (AUC) | | |
| --- | --- | --- | --- | --- | --- | --- |
|  | 12mo | 36mo | 60mo | 12mo | 36mo | 60mo |
| IMDC | 0.613 | 0.576 | 0.395 | 0.642 | 0.654 | 0.570 |
| LIPI | 0.620 | 0.574 | 0.575 | 0.628 | 0.622 | 0.546 |
| RMH | 0.599 | 0.583 | 0.418 | 0.644 | 0.644 | 0.606 |
| PMHI | 0.625 | 0.756 | 0.630 | 0.663 | 0.723 | 0.715 |
| GRIm | 0.593 | 0.578 | 0.417 | 0.634 | 0.636 | 0.530 |
| C-PLAN | 0.613 | 0.578 | 0.475 | 0.662 | 0.676 | 0.551 |
| mGPS | 0.578 | 0.533 | 0.385 | 0.590 | 0.622 | 0.556 |
| Meet-URO3 | 0.666 | 0.569 | 0.388 | 0.709 | 0.636 | 0.440 |

AUC: area under the curve; PFS: progression-free survival; OS: overall survival; IMDC: International Metastatic RCC Database Consortium; LIPI: Lung Immune Prognostic Index; RMH: Royal Marsden Hospital score; PMHI: Princess Margaret Hospital Index; GRIm: Gustave Roussy Immune Score; mGPS: modified Glasgow Prognostic Score.
